# Minimal Change Disease After First Dose of Pfizer-BioNTech COVID-19 Vaccine: A Case Report and Review of Minimal Change Disease Related to COVID-19 Vaccine

**DOI:** 10.1177/20543581211058271

**Published:** 2021-11-15

**Authors:** Jessica Hanna, Alistair Ingram, Tiffany Shao

**Affiliations:** 1 Department of Pathology and Molecular Medicine, McMaster University, St. Joseph’s Healthcare Hamilton, ON, Canada; 2 Department of Medicine, Division of Nephrology, McMaster University, St. Joseph’s Healthcare Hamilton, ON, Canada

**Keywords:** COVID-19 vaccine, minimal change disease, acute kidney injury, SARS-CoV-2

## Abstract

**Rationale:**

While severe complications are generally uncommon with novel coronavirus disease 2019 (COVID-19) vaccine, there has been a steady increase in the number of patients presenting with nephrotic syndrome and acute kidney injury after the administration of COVID-19 vaccine. Physicians should be made aware of minimal change disease as a potential complication associated with COVID-19 vaccine.

**Presenting concerns:**

A 60-year-old male without significant past medical history presented with new onset of nephrotic syndrome approximately 10 days after his first dose of Pfizer-BioNTech COVID-19 vaccine. Laboratory findings showed hypoalbuminemia (20 g/L), elevated urine albumin/creatinine ratio (668 mg/mmol), and elevated creatinine of 116 µmol/L from a baseline of 79 µmol/L.

**Diagnosis:**

A diagnostic kidney biopsy was performed 6 weeks after the onset of the edema and approximately 8 weeks after his first dose of Pfizer-BioNTech COVID-19 vaccine. The kidney biopsy findings were consistent with minimal change disease with focal acute tubular injury.

**Interventions:**

The patient was treated conservatively with ramipril 10 mg and furosemide 80 mg daily 5 weeks after the onset of swelling. Prednisone 1 mg/kg was initiated immediately when the kidney biopsy result became available (approximately 6 weeks after the onset of edema).

**Outcomes:**

The patient remitted with rapid weight loss starting 2 weeks post prednisone initiation.

**Novel findings:**

De novo minimal change disease with acute tubular injury is a kidney manifestation following the administration of Pfizer-BioNTech COVID-19 vaccine. Minimal change disease is potentially a rare complication of Pfizer-BioNTech COVID-19 vaccine.

## Introduction

The novel coronavirus disease 2019 (COVID-19) has caused significant mortality and morbidity worldwide. Several COVID-19 vaccines were developed and widely administered to combat this highly contagious disease. There has been a growing number of cases linking COVID-19 vaccines to an immunologic response resulting in the development of de novo minimal change disease (MCD).^1-7^

We report a case of a 60-year-old male who developed de novo MCD presenting with nephrotic syndrome and acute kidney injury (AKI) after his first dose of the Pfizer-BioNTech COVID-19 vaccine, an mRNA-based vaccine against severe acute respiratory syndrome coronavirus 2 (SARS CoV-2).

## Presenting Concerns

A 60-year-old male (smoker) without significant past medical history presented with new onset of nephrotic syndrome approximately 10 days after his first dose of Pfizer-BioNTech COVID-19 vaccine.

## Clinical Findings

The presenting symptom of ankle swelling began approximately 10 days after his initial dose of vaccine. He subsequently developed progressive fatigue and shortness of breath with exertion. Physical examination revealed bilateral crackles and bilateral peripheral pitting edema up to the abdomen. He was afebrile and hemodynamically stable with a blood pressure of 162/97 mm. His oxygen saturation was 98% on room air. The rest of the examination was unremarkable. He was not taking any medications, including nonsteroidal anti-inflammatory drugs.

## Diagnostic Focus and Assessment

Laboratory investigation 4 weeks after the onset of the swelling revealed an albumin of 24 g/L and urine albumin/creatinine ratio of 668 mg/mmol. He was seen by a nephrologist 5 weeks after the onset of the swelling, and creatinine had risen to 116 µmol/L from a baseline of 79 µmol/L. Urinalysis showed 3+ protein and 2+ blood. Laboratory investigation for antinuclear antibody (ANA), serum protein electrophoresis (SPEP), hepatitis B and C serology, and anti-phospholipase A2 receptor (PLA2R) were drawn the same day and subsequently returned negative. C3 and C4 were within normal limits. There was no evidence of infection, allergic exposure, or an underlying malignancy. Both prerenal and postrenal causes of AKI were excluded.

**Figure 1.**
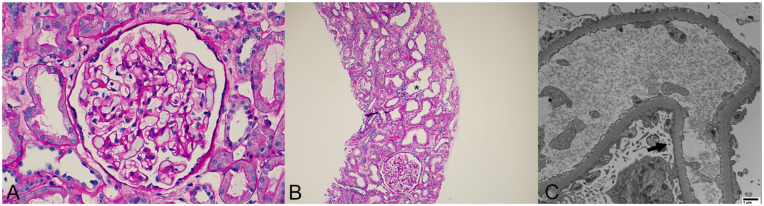
Kidney biopsy findings: (A) light microscopy shows a representative unremarkable glomerulus (periodic acid-Schiff stain, ×400), (B) area of acute tubular injury (asterisk, periodic acid-Schiff stain, ×100), and (C) electron microscopy reveals diffuse podocyte foot process effacement with microvillous transformation (arrow, ×10 000).

A diagnostic kidney biopsy was performed approximately 6 weeks after onset of the edema and 8 weeks after his first dose of Pfizer-BioNTech COVID-19 vaccine (Figure 1). Light microscopy revealed 10 patent glomeruli that were unremarkable. Some tubules showed acute tubular injurious changes characterized by attenuation of the tubular epithelium and variable loss of brush borders. There was focal mild tubular atrophy and interstitial fibrosis. Immunofluorescence microscopy was negative for any immune-complex deposition. Electron microscopy revealed diffuse podocyte foot process effacement with microvillous transformation. There were no electron-dense deposits. Overall, the findings were consistent with MCD with focal acute tubular injury.

## Therapeutic Focus and Assessment

He was started on ramipril 10 mg and furosemide 80 mg daily 5 weeks after the onset of swelling.

Prednisone 1 mg/kg (total 80 mg daily) was initiated 1 week later when the biopsy result was available.

## Follow-Up and Outcomes

**Figure 2.**
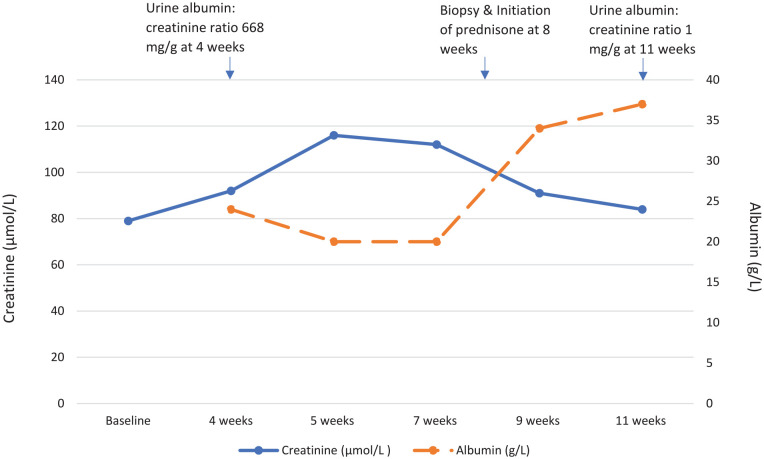
Temporal trends in serum creatinine (µmol/L), serum albumin (g/L), and proteinuria (urine albumin/creatinine ratio, mg/g) over the first 11 weeks after vaccination.

The patient remitted with rapid weight loss starting 14 days post prednisone initiation, with 70 pounds being lost over the subsequent week. Repeat investigations 3 weeks post kidney biopsy demonstrated preserved kidney function with a creatinine of 91 µmol/L, and an improvement of serum albumin from 20 to 34 g/L and an albumin/creatinine ratio of 1.0 mg/mmol (Figure 2). A follow-up urine albumin/creatinine ratio remained at 1.0 mg/mmol approximately 6 weeks post initiation of prednisone.

## Discussion

Recent reports have described new onset of MCD following vaccination for COVID-19^1-7^ (Table 1). We report a case of de novo MCD with AKI after the first dose of Pfizer-BioNTech COVID-19 vaccine.

**Table 1.** De Novo MCD Associated With Administration of COVID-19 Vaccine.

Reference	Country	Age	Sex	Vaccine	Dose	Onset of symptoms (days)	Presentation	Biopsy findings	Treatment	Outcome
D’Agati	The United States	77	Male	Pfizer	First	7	NS, AKI	MCD and ATI	S	PR, relapse after second dose
Lebedev	Israel	50	Male	Pfizer	First	4	NS, AKI	MCD and ATI	S	CR
Weijers	The Netherlands	61	Female	Pfizer	First	1	NS, AKI	MCD	S	PR
Leclerc	Canada	71	Male	AstraZeneca	First	13	NS, AKI	MCD and ATI	S	PR
Maas	The Netherlands	80s	Male	Pfizer	First	7	NS	MCD and ATI	S	CR
Holzworth	The United States	63	Female	Moderna	First	<7	NS, AKI	MCD, ATI, and AIN	S	Not reported
Salem	The United States	61	Female	Pfizer	Second	5	NS	MCD	S	Not reported

*Note.* Information adapted from references indicated and cited in the text. COVID-19 = coronavirus disease 2019; AKI = acute kidney injury; MCD = minimal change disease; ATI = acute tubular injury; S = steroids; PR = partial response; NA = not available; CR = complete response; AIN = acute interstitial nephritis; NS = nephrotic syndrome.

New onset of MCD has been reported following the influenza vaccine in the past.^8,9^ The temporal association between the development of MCD and vaccination suggests an underlying pathogenic association. The pathogenesis of COVID- or influenza-vaccine-associated development of MCD is not fully understood at present. The COVID-19 vaccines use different methods to elicit host immunity. Pfizer-BioNTech and Moderna vaccines employ a lipid nanoparticle complexed with nucleoside-modified mRNA that encodes the SARS-CoV-2 spike protein while the AstraZeneca vaccine employs an adenoviral vector which contains the gene sequence encoding the SARS-CoV-2 spike protein. The above vaccines are designed to induce the host to synthesize the COVID-19 spike protein, which in turn generates an effective immune response to the COVID-19 spike protein. The induced T-cell biased response includes an upregulation of the production of cytokines including interferon γ, tumor necrosis factor α, and interleukin 2 that can trigger podocytopathies and enhance B-cell production of immunoglobulins in predisposed patients.^10-12^ These cytokines may play a role in exacerbating subclinical or quiescent glomerular diseases via similar mechanism proposed for viral infections, which is a trigger for de novo and relapsing glomerular diseases.^13^

This case further supports the temporal association between COVID-19 vaccine (in this case Pfizer-BioNTech as with most of the previous reported cases) and the new onset of MCD in association with AKI. Acute kidney injury is a consistent feature reported in most of the cases.^1-5^ Although a causal association cannot be definitively confirmed, clinicians and pathologists should be aware of MCD presenting with nephrotic syndrome as a potential side effect. However, it is important to note that with the vast number of vaccines administered to date worldwide, only very rare occurrences of de novo MCD have been reported. A growing number of MCD cases relapsing after COVID-19 vaccination have been reported as well.^6,14-18^ Of note, not all patients with relapsed nephrotic syndrome post-COVID vaccine were biopsied and doubtless not all cases of de novo MCD have been reported in the literature.^19^ Therefore, the actual number of cases is likely much higher.

While the majority of the de novo MCD cases have been reported post Pfizer-BioNTech administration, cases associated with Moderna COVID-19 mRNA vaccination^2^ and with the non-mRNA-based AstraZeneca vaccine^4^ have also occurred. With prompt initiation of steroid therapy, complete remission of nephrotic syndrome and AKI can be achieved in the majority of cases. Relapse of MCD after the second dose of Pfizer-BioNTech has also been reported.^1,20^ Presently, there is as yet no guidance on how to proceed with the second dose of vaccine in such individuals. One potential solution is to switch to a different COVID-19 vaccine type to minimize the possibility of relapse. Importantly, more information is needed to guide optimal management of MCD as a potential complication post COVID-19 vaccine.
